# Association of *KRAS* and *NRAS* gene polymorphisms with Wilms tumor risk: a four-center case-control study

**DOI:** 10.18632/aging.101855

**Published:** 2019-03-12

**Authors:** Wen Fu, Zhenjian Zhuo, Rui-Xi Hua, Kai Fu, Wei Jia, Jinhong Zhu, Jiao Zhang, Jiwen Cheng, Haixia Zhou, Huimin Xia, Jing He, Guochang Liu

**Affiliations:** 1 Department of Pediatric Surgery, Guangzhou Institute of Pediatrics, Guangzhou Women and Children's Medical Center, Guangzhou Medical University, Guangzhou 510623, Guangdong, China; 2 School of Chinese Medicine, Faculty of Medicine, The Chinese University of Hong Kong, Hong Kong 999077, China; 3 Department of Oncology, The First Affiliated Hospital of Sun Yat-sen University, Guangzhou 510080, Guangdong, China; 4 Department of Clinical Laboratory, Molecular Epidemiology Laboratory, Harbin Medical University Cancer Hospital, Harbin 150040, Heilongjiang, China; 5 Department of Pediatric Surgery, The First Affiliated Hospital of Zhengzhou University, Zhengzhou 450052, Henan, China; 6 Department of Pediatric Surgery, The Second Affiliated Hospital of Xi'an Jiaotong University, Xi'an 710004, Shaanxi, China; 7 Department of Hematology, The Second Affiliated Hospital and Yuying Children's Hospital of Wenzhou Medical University, Wenzhou 325027, Zhejiang, China; * Equal contribution

**Keywords:** Wilms tumor, *KRAS*, *NRAS*, polymorphism, susceptibility

## Abstract

Wilms tumor is a type of pediatric solid tumor that arises partly due to somatic and germline mutations. Single-nucleotide polymorphisms (SNPs) in the *RAS* gene reportedly modify the risk for several types of human malignancies. We conducted a multicenter study to investigate whether *RAS* gene variants predispose individuals to Wilms tumor. Four SNPs in *RAS* were genotyped in 355 Wilms tumor cases and 1070 controls. The SNPs included rs12587 G>T, rs7973450 A>G and rs7312175 G>A in *KRAS*, and rs2273267 A>T in *NRAS*. Individuals harboring the rs12587 GT genotype were more likely to develop Wilms tumor than those carrying the GG genotype (adjusted odds ratio [OR]=1.30, 95% confidence interval [CI]=1.004-1.68, *P*=0.046). However, the other three SNPs seemed not to influence the risk for Wilms tumor. Compared to individuals without a risk genotype, those harboring one to three *KRAS* risk genotypes had an adjusted OR of 1.28 for developing Wilms tumor (95% CI=1.002-1.64, *P*=0.048). Stratification analysis revealed that rs12587 GT/TT was associated with Wilms tumor risk in children >18 months old (adjusted OR=1.39, 95% CI=1.02-1.89, *P*=0.037). Our findings indicate that the rs12587 G>T polymorphism in *KRAS* is associated with increased Wilms tumor susceptibility.

## Introduction

Wilms tumor (nephroblastoma) is the most common pediatric renal malignancy [1]. It is normally derived from embryonal kidney precursor cells in which cell growth and/or differentiation are dysregulated during development [2,3]. The incidence rate of Wilms tumor is about 1 in 10,000 children in Western countries [4]. The overall five-year survival rate exceeds 90% in developed countries [5–7]. Despite great achievements in the treatment of Wilms tumor, the outcomes for patients with high-risk disease (about 25%) remain disappointing [8]. Apart from this, high treatment costs and severe chronic health conditions that occur in nearly 25% of survivors are also challenging [9,10].

There is strong evidence that genetic factors contribute to Wilms tumor risk. To date, five Wilms tumor susceptibility loci have been well characterized, including Wilms tumor gene 1 (*WT1*), Wilms tumor gene on the X chromosome (*WTX*), catenin beta 1 (*CTNNB1*), tumor protein 53 (*TP53*) and the imprinted 11p15 region [11–13]. Although additional genetic variants continue to be identified, the carcinogenesis of Wilms tumor remains to be fully explained [14–16]. Therefore, it is indispensable to identify other genes that increase Wilms tumor susceptibility.

The *RAS* oncogene family has three members: *KRAS*, *NRAS* and *HRAS*. These genes encode a family of highly homologous GTPases that are involved in various cellular activities, such as growth, proliferation and differentiation [17,18]. *RAS* mutations have been detected in about 20% of human malignancies [19]. *KRAS* mutations are the most common, accounting for approximately 85% of all *RAS* mutations [20,21], followed by *NRAS* mutations (15%). *HRAS* mutations are very rare, constituting less than 1% of all *RAS* mutations [22].

The impact of *RAS* gene variants on the risk of cancer has been widely investigated, including in studies of colorectal cancer [23], lung cancer [24,25], breast cancer [26] and melanoma [27]. Clark et al. demonstrated that coordinated activation of *RAS* and β-catenin accelerated the growth and metastatic progression of Wilms tumor in a murine model [28]. They later reported that activating *KRAS* mutations were found in human Wilms tumor samples [29]. Recently, another team verified the importance of *RAS* mutations in the development and progression of Wilms tumor [30].

Despite these findings, the link between *RAS* gene polymorphisms and Wilms tumor risk remains obscure. To clarify the association of *RAS* with Wilms tumor risk, we selected single-nucleotide polymorphisms (SNPs) in the two most common diseased-related *RAS* genes, *KRAS* and *NRAS*, for analysis in a four-center hospital-based case-control study.

## RESULTS

### Correlation of *RAS* gene polymorphisms with Wilms tumor risk

We successfully genotyped 1070 controls and 351 cases for *KRAS* polymorphisms, along with 1070 controls and 355 cases for *NRAS* polymorphism. The demographic characteristics of the subjects are presented in Supplemental Table 1. All the SNP genotype frequencies were in Hardy-Weinberg equilibrium in controls (*P*>0.05). Our results indicated that the rs12587 GT genotype is a risk variant for Wilms tumor (Table 1), as individuals with this genotype had a 1.30-fold greater risk for developing Wilms tumor (95% confidence interval [CI]=1.004-1.68, *P*=0.046) than those with the GG genotype. The individual rs7973450 A>G, rs7312175 G>A and rs2273267 A>T variants did not predispose individuals to Wilms tumor.

**Table 1:** Logistic regression analysis of associations between *RAS* polymorphisms and Wilms tumor risk.

Genotype		Cases (N=355)	Controls (N=1070)	*P* ^a^	Crude OR (95% CI)	*P*	Adjusted OR (95% CI) ^b^	*P* ^b^
*KRAS* rs12587 G>T (HWE=0.287)								
GG		206 (58.69)	688 (64.30)		1.00		1.00	
GT		129 (36.75)	333 (31.12)		**1.29 (1.002-1.67)**	**0.049**	**1.30 (1.004-1.68)**	**0.046**
TT		16 (4.56)	49 (4.58)		1.09 (0.61-1.96)	0.772	1.08 (0.60-1.94)	0.806
Additive				0.142	1.18 (0.96-1.44)	0.117	1.18 (0.96-1.44)	0.120
Dominant		145 (41.31)	382 (35.70)	0.059	1.27 (0.99-1.62)	0.059	1.27 (0.99-1.63)	0.058
Recessive		335 (95.44)	1021 (95.42)	0.987	1.00 (0.56-1.77)	0.987	0.98 (0.55-1.75)	0.949
G		541 (77.07)	1709 (79.86)		1.00		1.00	
T		161 (22.93)	431 (20.14)	0.114	1.18 (0.96-1.45)	0.114	1.18 (0.96-1.45)	0.117
*KRAS* rs7973450 A>G (HWE=0.080)								
AA	282 (80.34)		881 (82.34)		1.00		1.00	
AG	68 (19.37)		185 (17.29)		1.15 (0.84-1.56)	0.380	1.14 (0.84-1.56)	0.402
GG	1 (0.28)		4 (0.37)		0.78 (0.09-7.02)	0.825	0.83 (0.09-7.50)	0.870
Additive				0.660	1.13 (0.84-1.52)	0.436	1.12 (0.83-1.51)	0.448
Dominant	69 (19.66)		189 (17.66)	0.400	1.14 (0.84-1.55)	0.401	1.14 (0.84-1.54)	0.418
Recessive	350 (99.72)		1066 (99.63)	0.807	0.76 (0.09-6.84)	0.808	0.81 (0.09-7.32)	0.853
A	632 (90.03)		1947 (90.98)		1.00		1.00	
G	70 (9.97)		193 (9.02)	0.450	1.12 (0.84-1.49)	0.450	1.11 (0.84-1.49)	0.462
*KRAS* rs7312175 G>A (HWE=0.130)								
GG		270 (76.92)	851 (79.53)		1.00		1.00	
GA		72 (20.51)	201 (18.79)		1.13 (0.84-1.53)	0.431	1.14 (0.84-1.54)	0.404
AA		9 (2.56)	18 (1.68)		1.58 (0.70-3.55)	0.272	1.54 (0.68-3.48)	0.298
Additive				0.423	1.17 (0.91-1.51)	0.222	1.17 (0.91-1.51)	0.218
Dominant		81 (23.08)	219 (20.47)	0.299	1.17 (0.87-1.56)	0.299	1.17 (0.88-1.57)	0.285
Recessive		342 (97.44)	1052 (98.32)	0.294	1.54 (0.69-3.46)	0.297	1.50 (0.67-3.39)	0.326
G		612 (87.18)	1903 (88.93)		1.00		1.00	
A		90 (12.82)	237 (11.07)	0.208	1.18 (0.91-1.53)	0.209	1.18 (0.91-1.53)	0.205
*NRAS* rs2273267 A>T (HWE=0.723)								
AA		183 (51.55)	541 (50.56)		1.00		1.00	
AT		142 (40.00)	443 (41.40)		0.95 (0.74-1.22)	0.676	0.95 (0.74-1.23)	0.714
TT		30 (8.45)	86 (8.04)		1.03 (0.66-1.61)	0.893	1.02 (0.65-1.61)	0.917
Additive				0.889	0.99 (0.82-1.19)	0.883	0.99 (0.82-1.19)	0.890
Dominant		172 (48.45)	529 (49.44)	0.747	0.96 (0.76-1.22)	0.747	0.97 (0.76-1.23)	0.774
Recessive		325 (91.55)	984 (91.96)	0.805	1.06 (0.68-1.63)	0.805	1.05 (0.68-1.62)	0.840
A		508 (71.55)	1525 (71.26)		1.00		1.00	
T		202 (28.45)	615 (28.74)	0.883	0.99 (0.82-1.19)	0.883	0.99 (0.82-1.19)	0.891
Combined effect of risk genotypes for *KRAS* ^c^								
0		200 (56.98)	673 (62.90)		1.00		1.00	
1		13 (3.70)	28 (2.62)		1.56 (0.80-3.07)	0.196	1.57 (0.80-3.10)	0.192
2		132 (37.61)	345 (32.24)		1.29 (1.00-1.66)	0.052	1.29 (1.00-1.66)	0.052
3		6 (1.71)	24 (2.24)		0.84 (0.34-2.09)	0.709	0.84 (0.34-2.09)	0.709
Trend				0.157	1.11 (0.98-1.25)	0.094	1.11 (0.98-1.25)	0.093
0		200 (56.98)	673 (62.90)		1.00		1.00	
1-3		151 (43.02)	397 (37.10)	0.048	**1.28 (1.002-1.64)**	**0.048**	**1.28 (1.002-1.64)**	**0.048**

OR, odds ratio; CI, confidence interval; HWE, Hardy-Weinberg equilibrium. ^a^ *χ^2^* test for genotype distributions between Wilms tumor patients and cancer-free controls. ^b^ Adjusted for age and gender. ^c^ Risk genotypes were carriers with rs12587 GT/TT, rs7973450 AG/GG and rs7312175 GA/AA genotypes.

We further examined the combined effects of the risk genotypes for *KRAS* on Wilms tumor risk. Compared to individuals without a risk genotype, those harboring one to three of these genotypes were at 1.28-fold greater risk for Wilms tumor (95% CI=1.002-1.64, *P*=0.048).

### Stratification analysis

Tables 2 and 3 summarize the analysis of *KRAS* and *NRAS* polymorphisms and Wilms tumor risk after stratification by age, gender and clinical stage. A significant association between rs12587 GT/TT and Wilms tumor risk was only found in children >18 months old among the analyzed strata (adjusted odds ratio [OR]=1.39, 95% CI=1.02-1.89, *P*=0.037).

**Table 2:** Stratification analysis for association between *KRAS* genotypes and Wilms tumor susceptibility.

Variables	rs12587 (case/control)		AOR (95% CI)^a^	*P*^a^	rs7973450 (case/control)		AOR (95% CI)^a^	*P*^a^	rs7312175 (case/control)		AOR (95% CI)^a^	*P*^a^	Combine genotypes (case/control)		AOR (95% CI)^a^	*P*^a^
	GG	GT/TT			AA	AG/GG			GG	GA/AA			0	1-3		
Age, month																
≤18	77/272	46/153	1.06 (0.70-1.61)	0.771	97/341	26/84	1.09 (0.67-1.79)	0.726	99/345	24/80	1.04 (0.63-1.74)	0.870	74/269	49/156	1.14 (0.76-1.73)	0.522
>18	129/416	99/229	**1.39 (1.02-1.89)**	**0.037**	185/540	43/105	1.20 (0.81-1.77)	0.373	171/506	57/139	1.21 (0.85-1.73)	0.286	126/404	102/241	1.35 (0.99-1.83)	0.056
Gender																
Female	97/283	66/165	1.17 (0.81-1.68)	0.412	128/369	35/79	1.28 (0.82-1.99)	0.285	129/354	34/94	0.99 (0.64-1.54)	0.973	93/277	70/171	1.22 (0.85-1.75)	0.287
Male	109/405	79/217	1.37 (0.98-1.92)	0.064	154/512	34/110	1.03 (0.67-1.57)	0.897	141/497	47/125	1.34 (0.91-1.97)	0.135	107/396	81/226	1.35 (0.96-1.88)	0.081
Clinical stages																
I	71/688	48/382	1.23 (0.84-1.82)	0.293	101/881	18/189	0.82 (0.48-1.38)	0.446	90/851	29/219	1.28 (0.82-2.00)	0.276	69/673	50/397	1.25 (0.85-1.84)	0.261
II	51/688	39/382	1.37 (0.89-2.13)	0.154	68/881	22/189	1.48 (0.89-2.45)	0.134	71/851	19/219	1.06 (0.62-1.80)	0.836	50/673	40/397	1.36 (0.88-2.09)	0.172
III	47/688	32/382	1.21 (0.76-1.93)	0.425	67/881	12/189	0.84 (0.44-1.58)	0.587	57/851	22/219	1.48 (0.88-2.47)	0.138	46/673	33/397	1.20 (0.75-1.91)	0.443
IV	28/688	17/382	1.08 (0.59-2.01)	0.797	34/881	11/189	1.51 (0.75-3.04)	0.246	39/851	6/219	0.59 (0.25-1.42)	0.241	27/673	18/397	1.12 (0.61-2.06)	0.714
I+II	122/688	87/382	1.29 (0.96-1.75)	0.096	169/881	40/189	1.08 (0.74-1.58)	0.698	161/851	48/219	1.19 (0.83-1.69)	0.350	119/673	90/397	1.30 (0.96-1.75)	0.093
III+IV	75/688	49/382	1.16 (0.79-1.71)	0.439	101/881	23/189	1.06 (0.66-1.72)	0.800	96/851	28/219	1.12 (0.72-1.75)	0.617	73/673	51/397	1.17 (0.80-1.71)	0.413

AOR, adjusted odds ratio; CI, confidence interval. ^a^ Adjusted for age and gender, omitting the corresponding stratify factor.

**Table 3:** Stratification analysis for the association between *NRAS* rs2273267 A>T polymorphism and Wilms tumor risk.

Variables	rs2273267 (cases/controls)		Crude OR	*P*	Adjusted OR ^a^	*P* ^a^
	AA	AT/TT	(95% CI)		(95% CI)	
Age, month						
≤18	58/199	67/226	1.02 (0.68-1.52)	0.934	1.01 (0.67-1.50)	0.975
>18	125/342	105/303	0.95 (0.70-1.28)	0.730	0.96 (0.71-1.30)	0.799
Gender						
Females	91/234	72/214	0.87 (0.60-1.24)	0.431	0.87 (0.60-1.24)	0.432
Males	92/307	100/315	1.06 (0.77-1.46)	0.727	1.05 (0.76-1.45)	0.764
Clinical stages						
I	67/541	52/529	0.79 (0.54-1.16)	0.235	0.80 (0.55-1.17)	0.252
II	41/541	51/529	1.27 (0.83-1.95)	0.271	1.27 (0.83-1.96)	0.269
III	41/541	38/529	0.95 (0.60-1.50)	0.819	0.95 (0.60-1.51)	0.832
IV	28/541	19/529	0.69 (0.38-1.26)	0.229	0.70 (0.38-1.26)	0.233
I+II	108/541	103/529	0.98 (0.73-1.31)	0.868	0.98 (0.73-1.32)	0.888
III+IV	69/541	57/529	0.85 (0.58-1.22)	0.373	0.85 (0.59-1.23)	0.392

OR, odds ratio; CI, confidence interval. ^a^ Adjusted for age and gender, omitting the corresponding stratify factor.

### False-positive report probability (FPRP) analysis

In FPRP analysis (Table 4), only at a prior probability level of 0.25 and an FPRP threshold of 0.2 did the increased Wilms tumor risk remain noteworthy in carriers of rs12587 GT (FPRP=0.141), children >18 months old with rs12587 GT/TT (FPRP=0.131) and those with one to three risk genotypes (FPRP=0.139).

**Table 4:** False-positive report probability analysis for the association between *KRAS* genotypes and Wilms tumor susceptibility.

Genotype	Crude OR (95% CI)	*P* ^a^	Statistical power ^b^	Prior probability				
				0.25	0.1	0.01	0.001	0.0001
rs12587 G>T								
GT vs. GG	1.29 (1.002-1.67)	0.049	0.886	**0.141**	0.330	0.844	0.982	0.998
GT/TT vs. GG								
>18 months	1.39 (1.02-1.89)	0.037	0.682	**0.131**	0.311	0.832	0.980	0.998
Risk genotypes								
1-3 vs. 0	1.28 (1.002-1.64)	0.048	0.903	**0.139**	0.326	0.841	0.982	0.998

OR, odds ratio; CI, confidence interval. ^a^ χ^2^ test was used to calculate the genotype frequency distributions. ^b^ Statistical power was calculated using the number of observations in the subgroup and the OR and *P* values in this table.

## DISCUSSION

Thus far, only a small portion of genetic loci have been found to increase the risk of Wilms tumor. This underscores the need to reveal more genetic loci that could predispose individuals to this disease. Herein, we evaluated the impact of *KRAS* and *NRAS* gene SNPs on the risk of Wilms tumor in 355 Wilms tumor patients and 1070 healthy control subjects. To the best of our knowledge, we are the first to report the association of *RAS* gene polymorphisms with Wilms tumor risk in Chinese children.

*KRAS* and *NRAS* have been mapped to chromosomes 12p12.1 and 1p13.2, respectively. Many studies have investigated the mechanisms by which *RAS* gene polymorphisms impact cancer risk. In particular, rs61764370 and rs712, two *KRAS* polymorphisms in miRNA-binding sites, have been intensively studied. These two SNPs are located in the 3’ untranslated region (UTR) of *KRAS*, where they disrupt a let-7 miRNA binding site, thus increasing *KRAS* expression and enhancing tumor growth [31]. Chin et al. studied 46 populations worldwide, and identified the rs61764370 SNP in the 3’ UTR of the *KRAS* gene (*KRAS*-LCS6). This SNP was associated with increased expression of *KRAS,* reduced expression of let-7 and increased risk of lung cancer [31]. Furthermore, this allele was demonstrated to elevate the risk of epithelial ovarian cancer [32] and triple-negative breast cancer [33]. In a population-based case-control study conducted in the US by Christensen et al., the *KRAS*-LCS6 variant genotype (rs61764370) was not associated with the overall risk of head and neck squamous cell carcinoma, but was associated with a significantly reduced survival time [34].

Wang et al. [7] reported that the rs712 polymorphism in the *KRAS* 3’ UTR was associated with a reduced risk for oral squamous cell carcinoma, while rs1137282 in *KRAS* exon 6 was not [35]. In contrast, in a study of 181 gastric cancer patients and 674 cancer-free controls, Li et al. found that the T allele of rs712 significantly enhanced the susceptibility to gastric cancer [36]. As different types of tissues and cells have different miRNA profiles, the effects of SNPs in specific 3’ UTRs may vary accordingly. Moreover, differences in the population sources, environmental exposures, sample sizes and selection criteria of subjects may also have influenced the contribution of *RAS* SNPs to cancer susceptibility of different types. Therefore, it is necessary to define the impact of *RAS* polymorphisms on the risk of a certain cancer type in a certain population.

Our findings indicated that carriers of the *KRAS* rs12587 GT genotype had a genetic predisposition to Wilms tumor risk. Unexpectedly, rs7973450 A>G, rs7312175 G>A and rs2273267 A>T were not significantly associated with Wilms tumor risk. The rs12587 G>T, rs7973450 A>G and rs2273267 A>T polymorphisms reside in different complementary miRNA sites. The different locations of these SNPs may be one reason for their different effects on cancer risk. Other plausible interpretations of the null association include the relatively small sample size and the low-penetrance susceptibility of single polymorphisms.

One limitation of this study was the relatively small sample size, which may have impaired the statistical power, especially for the stratification analysis. Another limitation was the restriction of the included population to a single ethnicity (Chinese Han), which may render the findings inapplicable to other populations. Further, though we analyzed four SNPs in the current study, additional SNPs should be considered in future studies. Lastly, the current study only focused on genetic factors, and gene-environment interaction analysis was not performed due to the lack of relevant information. Wilms tumor is a heterogeneous disease, and both genetic and environmental factors contribute to its tumorigenesis. Thus, more comprehensive studies are warranted.

In conclusion, this was the first multi-center evaluation of the association of *KRAS* and *NRAS* gene SNPs with Wilms tumor susceptibility. Our study has provided the first evidence that *KRAS* gene SNPs may increase Wilms tumor susceptibility. Ongoing epidemiological studies in other independent populations are warranted prior to extrapolation of the current conclusions.

## MATERIALS AND METHODS

### Study subjects

In total, 355 cases and 1070 healthy controls were included in this study (Supplemental Table 1). The subject selection criteria were described in detail in our previous study [37–43]. In brief, cases with newly diagnosed and histologically confirmed Wilms tumor were recruited from four centers in China (Guangzhou Women and Children’s Medical Center [37–43], The First Affiliated Hospital of Zhengzhou University, The Second Affiliated Hospital and Yuying Children's Hospital of Wenzhou Medical University, and The Second Affiliated Hospital of Xi'an Jiao Tong University). All the included cases were sporadic cases. The controls were healthy volunteers without a history of Wilms tumor, matched to the cases by age, gender and city of residency. All the subjects or their guardians provided written informed consent before participating. Approval of the study protocol was obtained from the Institutional Review Board of each center prior to the study.

### Polymorphism selection and genotyping

We analyzed three potential functional SNPs in the *KRAS* gene and one potential functional SNP in the *NRAS* gene. SNPs were selected from the NCBI dbSNP database (http://www.ncbi.nlm.nih.gov/projects/SNP) and SNPinfo (http://snpinfo.niehs.nih.gov/snpfunc.htm). These four SNPs could capture an additional 89 SNPs with R^2^ ≥ 0.8 (Supplemental Table 2). The selection criteria were set as previously described [42,44]. Genomic DNA was extracted from venous blood with a TIANamp Blood DNA Kit (TianGen Biotech Co. Ltd., Beijing, China). SNP genotyping was performed with a TaqMan SNP Genotyping Assay (Applied Biosystems, Foster City, CA, USA). Negative controls with water and 10% replicates were also genotyped to ensure genotyping accuracy. No discordant genotypes were found in the replicates.

### Statistical analysis

Statistical analysis was performed in SAS release 9.1 (SAS Institute, Cary, NC, USA). The genotype frequency distributions of the polymorphisms were first evaluated among the controls, and Hardy-Weinberg equilibrium was assessed with the χ^2^ test. The distribution of subject characteristics between cases and controls was examined with a two-sided χ^2^ test. Unadjusted and adjusted (for age and gender) ORs and 95% CIs were generated for both single and combined SNPs. We then determined the association of the SNPs with Wilms tumor risk using the OR and 95% CI calculated from multivariable logistic regression analysis. FPRP analysis was performed as described previously [45]. All results were considered statistically significant if *P*<0.05.

## SUPPLEMENTARY MATERIAL

Supplemental Table 1

Supplemental Table 2
