# Supplementary material for: Association of KRAS and NRAS gene polymorphisms with Wilms tumor risk: a four-center case-control study
Source: Aging (Albany NY). 2019 Mar 12;11(5):1551–63. doi: 10.18632/aging.101855 (PMC6428095; doi:10.18632/aging.101855)
Supplement: Supplemental Table 1 [file aging-11-101855-s001.doc]

| **Supplemental Table 1.** Frequency distribution of selected variables in **Wilms tumor patients and controls.** | | | | | |
| --- | --- | --- | --- | --- | --- |
| Variables | Cases (n=355) | | Controls (n=1070) | | *P^a^* |
|  | No. | % | No. | % |  |
| Age |  | |  | |  |
| Range, months | 1-148.63 | | 0.03-156 | | 0.131 |
| Mean ± SD, months | 30.67 ± 23.96 | | 32.27 ± 26.89 | |  |
| ≤18 months | 125 | 35.21 | 425 | 39.72 |  |
| >18 months | 230 | 64.79 | 645 | 60.28 |  |
| Gender |  |  |  |  | 0.182 |
| Female | 163 | 45.92 | 448 | 41.87 |  |
| Male | 192 | 54.08 | 622 | 58.13 |  |
| Clinical stages |  |  |  |  |  |
| I | 119 | 33.52 |  |  |  |
| II | 92 | 25.92 |  |  |  |
| III | 79 | 22.25 |  |  |  |
| IV | 47 | 13.24 |  |  |  |
| NA | 18 | 5.07 |  |  |  |
| SD, standard deviation; NA, not available.  ^a^ Two-sided *χ^2^* test for distributions between Wilms tumor patients and cancer-free controls. | | | | | |
