# Supplementary material for: Association of KRAS and NRAS gene polymorphisms with Wilms tumor risk: a four-center case-control study
Source: Aging (Albany NY). 2019 Mar 12;11(5):1551–63. doi: 10.18632/aging.101855 (PMC6428095; doi:10.18632/aging.101855)
Supplement: Supplemental Table 2 [file aging-11-101855-s002.doc]

| **Supplemental Table 2. SNPs captured by the four selected potentially functional SNPs as predicted by SNPinfo software.** | | | | | | | | | | | | | |
| --- | --- | --- | --- | --- | --- | --- | --- | --- | --- | --- | --- | --- | --- |
| **rs** | **Chr.** | **Allele** | **LDsnp** | **Pop/LD** | **TFBS** | **Splicing**  **(ESE or ESS)** | **miRNA**  **(miRanda)** | **nsSNP** | **Nearby Gene** | **Distance (bp)** | **Allele** | **Asian** | **CHB** |
| rs10842466 | 12 | A/G | rs12587 | CHB/0.856 | -- | -- | -- | -- | *LRMP* | 46140||9888 | G | 0.217 | 0.274 |
| rs10842492 | 12 | G/T | rs12587 | CHB/0.818 | -- | -- | -- | -- | *CASC1* | 45785||41086 | T | 0.237 | 0.287 |
| rs10842494 | 12 | C/T | rs12587 | CHB/0.842 | -- | -- | -- | -- | *CASC1* | 48236||38635 | T | 0.767 | 0.700 |
| rs10842496 | 12 | G/T | rs12587 | CHB/0.831 | -- | Y | -- | Y | *CASC1* | 50266||36605 | G | 0.758 | 0.720 |
| rs10842498 | 12 | C/T | rs12587 | CHB/1 | -- | -- | -- | -- | *CASC1* | 76131||10740 | C | 0.225 | 0.267 |
| rs10842501 | 12 | C/T | rs12587 | CHB/1 | Y | -- | -- | -- | *CASC1* | 82293||4578 | T | 0.781 | 0.756 |
| rs10842502 | 12 | C/T | rs12587 | CHB/0.941 | Y | -- | -- | -- | *CASC1* | 82484||4387 | T | 0.762 | 0.716 |
| rs10842505 | 12 | A/G | rs12587 | CHB/1 | -- | -- | -- | -- | *LYRM5* | 5442||4357 | A | 0.791 | 0.731 |
| rs11047824 | 12 | A/G | rs12587 | CHB/0.887 | -- | -- | -- | -- | *LRMP* | 40512||15516 | G | 0.204 | 0.273 |
| rs11047865 | 12 | C/G | rs12587 | CHB/0.833 | -- | -- | -- | -- | *CASC1* | 45284||41587 | C | 0.236 | 0.284 |
| rs11047887 | 12 | A/C | rs12587 | CHB/1 | Y | -- | -- | -- | *LYRM5* | 522||9277 | A | 0.222 | 0.244 |
| rs11047888 | 12 | C/T | rs12587 | CHB/1 | Y | -- | -- | -- | *LYRM5* | 666||9133 | T | 0.778 | 0.756 |
| rs11047894 | 12 | C/G | rs12587 | CHB/1 | -- | -- | -- | -- | *KRAS* | 7495||38179 | C | 0.778 | 0.733 |
| rs11047901 | 12 | A/G | rs12587 | CHB/1 | -- | -- | -- | -- | *KRAS* | 18149||27525 | A | 0.219 | 0.267 |
| rs11047902 | 12 | C/T | rs12587 | CHB/1 | -- | -- | -- | -- | *KRAS* | 21613||24061 | C | 0.193 | 0.267 |
| rs1137188 | 12 | G/A | rs12587 | CHB/1 | -- | -- | Y | -- | *KRAS* | 1172||44502 | A | 0.778 | 0.727 |
| rs11611468 | 12 | A/C | rs12587 | CHB/1 | -- | -- | -- | -- | *CASC1* | 79900||6971 | C | 0.785 | 0.757 |
| rs11832421 | 12 | C/T | rs12587 | CHB/0.831 | -- | -- | -- | -- | *LRMP* | 42465||13563 | T | 0.787 | 0.720 |
| rs12368504 | 12 | C/T | rs12587 | CHB/1 | -- | -- | -- | -- | *KRAS* | 19512||26162 | T | 0.772 | 0.756 |
| rs12423443 | 12 | C/T | rs12587 | CHB/0.807 | -- | -- | -- | -- | *CASC1* | 69228||17643 | T | -- | 0.714 |
| rs12579073 | 12 | A/C | rs12587 | CHB/1 | -- | -- | -- | -- | *KRAS* | 17619||28055 | C | 0.116 | 0.244 |
| rs12579942 | 12 | C/T | rs12587 | CHB/1 | -- | -- | -- | -- | *KRAS* | 25014||20660 | T | 0.810 | 0.756 |
| **rs12587** | **12** | **T/G** | **rs12587** | **1** | **--** | **--** | **Y** | **--** | ***KRAS*** | **648||45026** | **G** | **0.807** | **0.756** |
| rs12810577 | 12 | A/G | rs12587 | CHB/0.91 | Y | -- | -- | -- | *CASC1* | 84940||1931 | G | 0.222 | 0.262 |
| rs12815546 | 12 | C/T | rs12587 | CHB/1 | -- | -- | -- | -- | *KRAS* | 24362||21312 | T | 0.778 | 0.756 |
| rs12822857 | 12 | A/G | rs12587 | CHB/1 | -- | -- | -- | -- | *KRAS* | 11437||34237 | G | 0.775 | 0.727 |
| rs13096 | 12 | T/C | rs12587 | CHB/1 | -- | -- | Y | -- | *KRAS* | 1661||44013 | T | 0.190 | 0.244 |
| rs17329025 | 12 | A/G | rs12587 | CHB/0.91 | -- | -- | -- | -- | *KRAS* | 25633||20041 | A | 0.193 | 0.262 |
| rs1908946 | 12 | G/C | rs12587 | CHB/0.891 | -- | -- | -- | Y | *LRMP* | 37874||18154 | G | 0.214 | 0.278 |
| rs2352782 | 12 | G/A | rs12587 | CHB/0.806 | -- | -- | -- | -- | *CASC1* | 24735||62136 | G | 0.219 | 0.289 |
| rs4246229 | 12 | A/G | rs12587 | CHB/1 | -- | -- | -- | -- | *KRAS* | 9489||36185 | A | 0.807 | 0.759 |
| rs4963859 | 12 | A/C | rs12587 | CHB/1 | -- | -- | -- | -- | *KRAS* | 9658||36016 | C | 0.785 | 0.733 |
| rs4963860 | 12 | C/T | rs12587 | CHB/1 | -- | -- | -- | -- | *KRAS* | 14015||31659 | C | 0.778 | 0.733 |
| rs712 | 12 | C/A | rs12587 | CHB/1 | -- | -- | Y | -- | *KRAS* | 4372||41302 | A | 0.193 | 0.250 |
| rs7299998 | 12 | C/T | rs12587 | CHB/0.882 | -- | -- | -- | -- | *CASC1* | 70161||16710 | T | 0.864 | 0.732 |
| rs7302922 | 12 | C/T | rs12587 | CHB/1 | Y | -- | -- | -- | *LYRM5* | 1167||8632 | T | 0.778 | 0.756 |
| rs7306769 | 12 | A/G | rs12587 | CHB/0.856 | -- | -- | -- | -- | *CASC1* | 53831||33040 | A | 0.758 | 0.726 |
| rs7308865 | 12 | A/C | rs12587 | CHB/0.841 | -- | -- | -- | -- | *CASC1* | 49501||37370 | C | 0.779 | 0.693 |
| rs9266 | 12 | G/A | rs12587 | CHB/1 | -- | -- | Y | -- | *KRAS* | 4037||41637 | G | 0.811 | 0.756 |
| rs9634100 | 12 | C/T | rs12587 | CHB/0.837 | -- | -- | -- | -- | *CASC1* | 51646||35225 | C | 0.759 | 0.705 |
| **rs2273267** | **1** | **T/A** | **rs2273267** | **1** | **Y** | **Y** | **--** | **--** | ***NRAS*** | **9884||46** | **A** | **0.720** | **0.816** |
| rs10842508 | 12 | C/T | rs7312175 | CHB/0.956 | -- | -- | -- | -- | *KRAS* | 13282||32392 | C | 0.900 | 0.839 |
| rs10842509 | 12 | C/G | rs7312175 | CHB/0.92 | -- | -- | -- | -- | *KRAS* | 17766||27908 | G | 0.889 | 0.833 |
| rs11047826 | 12 | C/T | rs7312175 | CHB/0.848 | -- | -- | -- | -- | *LRMP* | 42929||13099 | T | 0.884 | 0.805 |
| rs11047880 | 12 | A/G | rs7312175 | CHB/1 | -- | -- | -- | -- | *CASC1* | 78620||8251 | A | 0.909 | 0.866 |
| rs11047882 | 12 | C/T | rs7312175 | CHB/0.956 | -- | -- | -- | -- | *CASC1* | 79954||6917 | T | 0.898 | 0.839 |
| rs11047918 | 12 | A/G | rs7312175 | CHB/1 | -- | -- | -- | -- | *KRAS* | 38852||6822 | G | 0.873 | 0.844 |
| rs12228277 | 12 | A/T | rs7312175 | CHB/0.919 | -- | -- | -- | -- | *KRAS* | 39838||5836 | T | 0.908 | 0.812 |
| rs12229161 | 12 | C/T | rs7312175 | CHB/0.809 | -- | -- | -- | -- | *LRMP* | 43583||12445 | C | 0.888 | 0.815 |
| rs12230737 | 12 | A/G | rs7312175 | CHB/1 | -- | -- | -- | -- | *KRAS* | 30531||15143 | G | 0.887 | 0.788 |
| rs12423489 | 12 | C/T | rs7312175 | CHB/1 | -- | -- | -- | -- | *CASC1* | 80819||6052 | T | 0.899 | 0.841 |
| rs12424283 | 12 | A/G | rs7312175 | CHB/0.92 | -- | -- | -- | -- | *KRAS* | 19846||25828 | A | 0.875 | 0.814 |
| rs12427141 | 12 | A/G | rs7312175 | CHB/1 | -- | -- | -- | -- | *KRAS* | 8625||37049 | G | 0.904 | 0.844 |
| rs2970532 | 12 | C/T | rs7312175 | CHB/0.956 | -- | -- | -- | -- | *KRAS* | 37855||7819 | C | 0.898 | 0.839 |
| rs3782188 | 12 | A/G | rs7312175 | CHB/0.85 | -- | -- | -- | -- | *LRMP* | 44304||11724 | A | 0.893 | 0.818 |
| rs3924649 | 12 | G/A | rs7312175 | CHB/0.842 | -- | -- | -- | -- | *CASC1* | 77051||9820 | A | 0.908 | 0.821 |
| rs4623993 | 12 | C/T | rs7312175 | CHB/1 | -- | -- | -- | -- | *KRAS* | 27248||18426 | C | 0.952 | 0.844 |
| **rs7312175** | **12** | **A/G** | **rs7312175** | **1** | **Y** | **--** | **--** | **--** | ***KRAS||LOC100133222*** | **-750||-157604** | **G** | **0.906** | **0.845** |
| rs7973746 | 12 | C/G | rs7312175 | CHB/1 | -- | -- | -- | -- | *KRAS* | 33059||12615 | G | 0.916 | 0.845 |
| rs7979296 | 12 | G/T | rs7312175 | CHB/0.956 | Y | -- | -- | -- | *LYRM5* | 364||9435 | G | 0.928 | 0.839 |
| rs10505959 | 12 | C/T | rs7973450 | CHB/1 | -- | -- | -- | -- | *LRMP* | 49335||6693 | C | 0.884 | 0.917 |
| rs10771166 | 12 | C/T | rs7973450 | CHB/1 | -- | -- | -- | -- | *LRMP* | 44544||11484 | C | 0.899 | 0.909 |
| rs10771174 | 12 | C/T | rs7973450 | CHB/0.887 | -- | -- | -- | -- | *CASC1* | 46348||40523 | C | 0.853 | 0.869 |
| rs10771175 | 12 | C/G | rs7973450 | CHB/1 | -- | -- | -- | -- | *CASC1* | 51425||35446 | G | 0.882 | 0.898 |
| rs10771176 | 12 | C/T | rs7973450 | CHB/1 | -- | -- | -- | -- | *CASC1* | 51497||35374 | T | 0.876 | 0.917 |
| rs10842464 | 12 | C/T | rs7973450 | CHB/1 | -- | -- | -- | -- | *LRMP* | 44502||11526 | T | 0.101 | 0.091 |
| rs10842470 | 12 | G/T | rs7973450 | CHB/1 | -- | -- | -- | -- | *CASC1* | 4980||81891 | T | 0.888 | 0.917 |
| rs10842490 | 12 | C/G | rs7973450 | CHB/1 | -- | -- | -- | -- | *CASC1* | 45380||41491 | C | 0.872 | 0.868 |
| rs11047825 | 12 | C/T | rs7973450 | CHB/0.877 | -- | -- | -- | -- | *LRMP* | 41847||14181 | T | 0.895 | 0.907 |
| rs11047858 | 12 | C/T | rs7973450 | CHB/0.887 | -- | -- | -- | -- | *CASC1* | 29567||57304 | T | 0.867 | 0.878 |
| rs11047885 | 12 | A/C | rs7973450 | CHB/1 | -- | -- | -- | -- | *CASC1* | 81407||5464 | A | 0.891 | 0.917 |
| rs11834088 | 12 | G/T | rs7973450 | CHB/1 | -- | -- | -- | -- | *LRMP* | 40930||15098 | G | 0.893 | 0.909 |
| rs12227966 | 12 | G/T | rs7973450 | CHB/1 | -- | -- | -- | -- | *CASC1* | 44450||42421 | G | 0.895 | 0.893 |
| rs12228638 | 12 | A/G | rs7973450 | CHB/0.807 | -- | -- | -- | -- | *CASC1* | 21281||65590 | G | 0.101 | 0.101 |
| rs12367971 | 12 | A/G | rs7973450 | CHB/1 | -- | -- | -- | -- | *CASC1* | 50110||36761 | G | 0.886 | 0.917 |
| rs1497253 | 12 | G/A | rs7973450 | CHB/0.888 | -- | -- | -- | -- | *LRMP* | 40807||15221 | A | 0.894 | 0.884 |
| rs2220196 | 12 | T/G | rs7973450 | CHB/1 | -- | -- | -- | -- | *CASC1* | 23249||63622 | G | 0.903 | 0.900 |
| rs3924650 | 12 | T/C | rs7973450 | CHB/1 | -- | -- | -- | -- | *CASC1* | 77006||9865 | T | 0.907 | 0.895 |
| rs4313666 | 12 | A/G | rs7973450 | CHB/1 | -- | -- | -- | -- | *CASC1* | 43484||43387 | A | 0.878 | 0.889 |
| rs7134616 | 12 | C/G | rs7973450 | CHB/1 | -- | -- | -- | -- | *CASC1* | 2667||84204 | G | 0.888 | 0.916 |
| rs7303373 | 12 | A/T | rs7973450 | CHB/1 | -- | -- | -- | -- | *CASC1* | 53583||33288 | A | 0.878 | 0.889 |
| rs7303669 | 12 | C/T | rs7973450 | CHB/0.927 | -- | -- | -- | -- | *LRMP* | 40363||15665 | T | 0.894 | 0.910 |
| rs7960092 | 12 | A/C | rs7973450 | CHB/0.807 | -- | -- | -- | -- | *LRMP* | 46937||9091 | C | 0.889 | 0.899 |
| rs7960428 | 12 | A/G | rs7973450 | CHB/1 | -- | -- | -- | -- | *LRMP* | 47047||8981 | G | 0.889 | 0.893 |
| rs7960917 | 12 | C/T | rs7973450 | CHB/1 | -- | -- | Y | -- | *KRAS* | 3466||42208 | T | 0.882 | 0.917 |
| rs7964195 | 12 | C/T | rs7973450 | CHB/1 | -- | -- | -- | -- | *LRMP* | 38938||17090 | T | 0.893 | 0.909 |
| rs7971062 | 12 | C/T | rs7973450 | CHB/1 | -- | -- | -- | -- | *CASC1* | 36490||50381 | T | 0.888 | 0.898 |
| **rs7973450** | **12** | **A/G** | **rs7973450** | **1** | **--** | **--** | **Y** | **--** | ***KRAS*** | **2962||42712** | **A** | **--** | **0.917** |
| rs7975271 | 12 | C/T | rs7973450 | CHB/0.927 | -- | -- | -- | -- | *LRMP* | 47169||8859 | T | 0.907 | 0.911 |
| rs7976254 | 12 | C/T | rs7973450 | CHB/1 | -- | -- | -- | -- | *LRMP* | 51113||4915 | C | 0.878 | 0.916 |
| rs7977670 | 12 | A/G | rs7973450 | CHB/0.927 | -- | -- | -- | -- | *LRMP* | 39142||16886 | A | 0.889 | 0.911 |
| rs7980769 | 12 | C/T | rs7973450 | CHB/1 | -- | -- | -- | -- | *CASC1* | 77720||9151 | T | 0.878 | 0.917 |
| SNP, single nucleotide polymorphism; LD, linkage disequilibrium; TFBS, transcription factor binding sites; ESE, exonic splicing enhancer; ESS, exonic splicing silencer; nsSNP, nonsynonymous single nucleotide polymorphism; CHB, Han Chinese in Beijing, China. | | | | | | | | | | | | | |
